# On a Cubically Convergent Iterative Method for Matrix Sign

**DOI:** 10.1155/2015/964257

**Published:** 2015-03-29

**Authors:** M. Sharifi, S. Karimi Vanani, F. Khaksar Haghani, M. Arab, S. Shateyi

**Affiliations:** ^1^Department of Mathematics, Islamic Azad University, Shahrekord Branch, Shahrekord, Iran; ^2^Department of Mathematics and Applied Mathematics, University of Venda, Thohoyandou 0950, South Africa

## Abstract

We propose an iterative method for finding matrix sign function. It is shown that the scheme has global behavior with cubical rate of convergence. Examples are included to show the applicability and efficiency of the proposed scheme and its reciprocal.

## 1. Introduction

It is known that the function of sign in the scalar case is defined for any *z* ∈ *ℂ* not on the imaginary axis by

(1) signz=1,Rez>0,−1,Rez<0.  

An extension of (1) for the matrix case was given firstly by Roberts in [1]. This extended matrix function is of clear importance in several applications (see, e.g., [2] and the references therein).

Assume that *A* ∈ *ℂ* ^*n*×*n*^ is a matrix with no eigenvalues on the imaginary axis. To define this matrix function formally, let

(2) $$\begin{array}{c} A=TJT^{-1} \end{array}$$

be a Jordan canonical form arranged so that *J* = diag(*J*_1_, *J*_2_), where the eigenvalues of *J*_1_ ∈ *ℂ* ^*p*×*p*^ lie in the open left half-plane and those of *J*_2_ ∈ *ℂ* ^*q*×*q*^ lie in the open right half-plane; then

(3) $$\begin{array}{c} S=\mathrm{sign}\left(A\right)=T\left(\begin{array}{cc} -I_{p} & 0\\ 0 & I_{q} \end{array}\right)T^{-1}, \end{array}$$

where *p* + *q* = *n*. A simplified definition of the matrix sign function for Hermitian case (eigenvalues are all real) is

(4) $$\begin{array}{c} S=U\mathrm{diag}\left(\mathrm{sign}\left(\lambda_{1}\right),\ldots ,\mathrm{sign}\left(\lambda_{n}\right)\right)U^{*}, \end{array}$$

where

(5) $$\begin{array}{c} U^{*}AU=\mathrm{diag}\left(\lambda_{1},\ldots ,\lambda_{n}\right) \end{array}$$

is a diagonalization of *A*.

The importance of computing *S* is also due to the fact that the sign function plays a fundamental role in iterative methods for matrix roots and the polar decomposition [3].

Note that although sign(*A*) is a square root of the identity matrix, it is not equal to *I* or −*I* unless the spectrum of *A* lies entirely in the open right half-plane or open left half-plane, respectively. Hence, in general, sign(*A*) is a nonprimary square root of *I*.

In this paper, we focus on iterative methods for finding *S*. In fact, such methods are Newton-type schemes which are in essence fixed-point-type methods by producing a convergent sequence of matrices via applying a suitable initial matrix.

The most famous method of this class is the quadratic Newton method defined by

(6) $$\begin{array}{c} X_{k+1}=\frac{1}{2}\left(X_{k}+X_{k}^{-1}\right). \end{array}$$

It should be remarked that iterative methods, such as (6), and the Newton-Schultz iteration

(7) $$\begin{array}{c} X_{k+1}=\frac{1}{2}X_{k}\left(3I-X_{k}^{2}\right) \end{array}$$

or the cubically convergent Halley method

(8) $$\begin{array}{c} X_{k+1}=\left[I+3X_{k}^{2}\right]{\left[X_{k}\left(3I+X_{k}^{2}\right)\right]}^{-1}, \end{array}$$

are all special cases of the Padé family proposed originally in [4]. The Padé approximation belongs to a broader category of rational approximations. Coincidentally, the best uniform approximation of the sign function on a pair of symmetric but disjoint intervals can be expressed as a rational function.

Note that although (7) does not possess a global convergence behavior, on state-of-the-art parallel computer architectures, matrix inversions scale less satisfactorily than matrix multiplications do, and subsequently (7) is useful in some problems. However, due to local convergence behavior, it is excluded from our numerical examples in this work.

The rest of this paper is organized as follows. In Section 2, we discuss how to construct a new iterative method for finding (3). It is also shown that the constructed method is convergent with cubical rate. It is noted that its reciprocal iteration obtained from our main method is also convergent. Numerical examples are furnished to show the higher numerical accuracy for the constructed solvers in Section 3. The paper ends in Section 4 with some concluding comments.

## 2. A New Method

The connection of matrix iteration methods with the sign function is not immediately obvious, but in fact such methods can be derived by applying a suitable root-finding method to the nonlinear matrix equation

(9) $$\begin{array}{c} X^{2}=I \end{array}$$

and when of course sign(*A*) is one solution of this equation (see for more [5]).

Here, we consider the following root-solver:

(10) $$\begin{array}{c} x_{k+1}=x_{k}-\frac{10-4L\left(x_{k}\right)}{10-9L\left(x_{k}\right)}\frac{f\left(x_{k}\right)}{f^{\prime }\left(x_{k}\right)}, \end{array}$$

with *L*(*x*_*k*_) = *f*′′(*x*_*k*_)*f*(*x*_*k*_)/*f*′(*x*_*k*_)^2^. In what follows, we observe that (10) possesses third order of convergence.

**Theorem 1 .**

Let *α* ∈ *D* be a simple zero of a sufficiently differentiable function *f* : *D*⊆*ℂ* → *ℂ*, which contains *x*_0_ as an initial approximation. Then the iterative expression (10) satisfies

(11) $$\begin{array}{c} e_{k+1}=\left(\frac{c_{2}^{2}}{5}-c_{3}\right)e_{k}^{3}+O\left(e_{k}^{4}\right), \end{array}$$

where *c*_*j*_ = *f* ^(*j*)^(*α*)/*j*!*f*′(*α*), *e*_*k*_ = *x*_*k*_ − *α*.

**Proof**

The proof would be similar to the proofs given in [6].

Applying (10) on the matrix equation (9) will result in the following new matrix fixed-point-type iteration for finding (3):

(12) $$\begin{array}{c} X_{k+1}=\left(2I+15X_{k}^{2}+3X_{k}^{4}\right){\left[9X_{k}+11X_{k}^{3}\right]}^{-1}, \end{array}$$

where *X*_0_ = *A*. This is named PM1 from now on.

**Figure 1:**
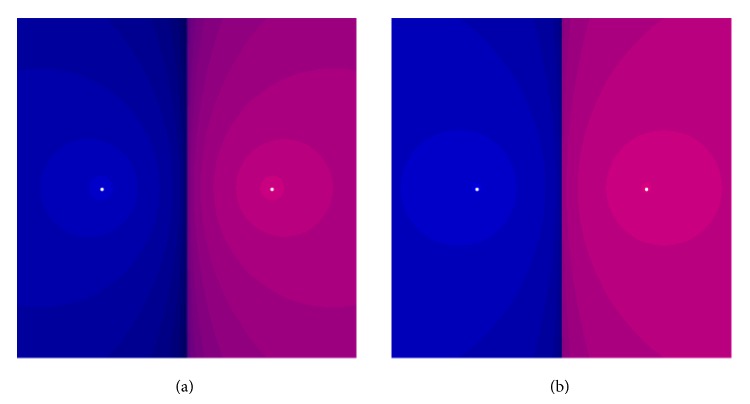
Attraction basins for (6) (a) and (8) (b) for the polynomial *g*(*x*) = *x* ^2^ − 1.

**Figure 2:**
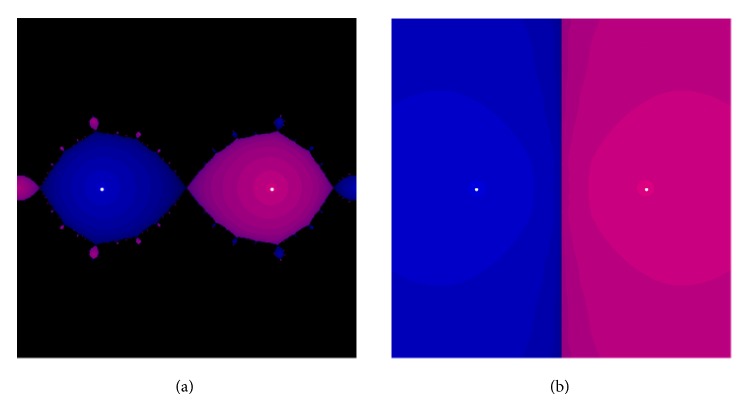
Attraction basins of (7) (a) and (12) (b) for the polynomial *g*(*x*) = *x* ^2^ − 1.

The proposed scheme (12) is not a member of Padé family [4]. Furthermore, applying (10) on the scalar equation *g*(*x*) = *x* ^2^ − 1 provides a global convergence in the complex plane (except the points lying on the imaginary axis). This global behavior, which is kept for matrix case, has been illustrated in Figure 1 by drawing the basins of attraction for (6) and (8). The attraction basins for (7) (local convergence) and (12) (global convergence) are also portrayed in Figure 2.

**Theorem 2 .**

Let *A* ∈ *ℂ* ^*n*×*n*^ have no pure imaginary eigenvalues. Then, the matrix sequence {*X*_*k*_}_*k*=0_^*k*=*∞*^ defined by (12) converges to *S*, choosing *X*_0_ = *A*.

**Proof**

We remark that all matrices, whether they are diagonalizable or not, have a Jordan normal form *A* = *TJT* ^−1^, where the matrix *J* consists of Jordan blocks. For this reason, let *A* have a Jordan canonical form arranged as

(13) $$\begin{array}{c} T^{-1}AT=\Lambda =\left[\begin{array}{cc} C & 0\\ 0 & N \end{array}\right], \end{array}$$

where *T* is a nonsingular matrix and *C*, *N* are square Jordan blocks corresponding to eigenvalues lying in *ℂ* ^−^ and *ℂ* ^+^, respectively. We have

(14) signΛ =signT−1AT=T−1signAT =diagsignλ1,…,signλp,signλp+1,…,signλn.

If we define *D*_*k*_ = *T* ^−1^ *X*_*k*_ *T*, then, from the method (12), we obtain

(15) $$\begin{array}{c} D_{k+1}=\left(2I+15D_{k}^{2}+3D_{k}^{4}\right){\left[9D_{k}+11D_{k}^{3}\right]}^{-1}. \end{array}$$

Note that if *D*_0_ is a diagonal matrix, then, based on an inductive proof, all successive *D*_*k*_ are diagonal too. From (15), it is enough to show that {*D*_*k*_} converges to sign(Λ). We remark that the case at which *D*_0_ is not diagonal will be discussed later in the proof.

In the meantime, we can write (15) as *n* uncoupled scalar iterations to solve *g*(*x*) = *x* ^2^ − 1 = 0, given by

(16) $$\begin{array}{c} d_{k+1}^{i}=\left(2+15{d_{k}^{i}}^{2}+3{d_{k}^{i}}^{4}\right){\left[9d_{k}^{i}+11{d_{k}^{i}}^{3}\right]}^{-1}, \end{array}$$

where *d*_*k*_^*i*^ = (*D*_*k*_)_*i*,*i*_ and 1 ≤ *i* ≤ *n*. From (15) and (16), it is enough to study the convergence of {*d*_*k*_^*i*^} to sign(*λ*_*i*_).

It is known that sign(*λ*_*i*_) = *s*_*i*_ = ±1. Thus, we attain

(17) $$\begin{array}{c} \frac{d_{k+1}^{i}-1}{d_{k+1}^{i}+1}=\frac{{\left(-1+d_{k}^{i}\right)}^{3}\left(-2+3d_{k}^{i}\right)}{{\left(1+d_{k}^{i}\right)}^{3}\left(2+3d_{k}^{i}\right)}. \end{array}$$

Since |*d*_0_^*i*^ | = |*λ*_*i*_ | >0, we have

(18) $$\begin{array}{c} \underset{k\to \infty }{\lim}\left|\frac{d_{k+1}^{i}-1}{d_{k+1}^{i}+1}\right|=0, \end{array}$$

and lim_*k*→*∞*_ | *d*_*k*_^*i*^ | = 1 = |sign(*λ*_*i*_)|. This shows that {*d*_*k*_^*i*^} is convergent.

In the convergence proof, *D*_0_ may not be diagonal. Since the Jordan canonical form of some matrices may not be diagonal, thus, one cannot write (15) as *n* uncoupled scalar iterations (16). We comment that in this case our method is also convergent. To this goal, we must pursue the scalar relationship among the eigenvalues of the iterates for the studied rational matrix iteration.

In this case, the eigenvalues of *X*_*k*_ are mapped from the iterate *k* to the iterate *k* + 1 by the following relation:

(19) $$\begin{array}{c} \lambda_{k+1}^{i}=\left(2+15{\lambda_{k}^{i}}^{2}+3{\lambda_{k}^{i}}^{4}\right){\left[9\lambda_{k}^{i}+11{\lambda_{k}^{i}}^{3}\right]}^{-1}. \end{array}$$

So, (19) clearly shows that the eigenvalues in the general case are convergent to ±1; that is to say,

(20) $$\begin{array}{c} \underset{k\to \infty }{\lim}\left|\frac{\lambda_{k+1}^{i}-1}{\lambda_{k+1}^{i}+1}\right|=0. \end{array}$$

Consequently, we have

(21) $$\begin{array}{c} \underset{k\to \infty }{\lim}X_{k}=T\left(\underset{k\to \infty }{\lim}D_{k}\right)T^{-1}=T\mathrm{sign}\left(\Lambda \right)\;T^{-1}=\mathrm{sign}\left(A\right). \end{array}$$

The proof is ended.

**Theorem 3 .**

Let *A* ∈ *ℂ* ^*n*×*n*^ have no pure imaginary eigenvalues. Then the proposed method (12) converges cubically to the sign matrix *S*.

**Proof**

Clearly, *X*_*k*_ are rational functions of *A* and, hence, like *A*, commute with *S*. On the other hand, we know that *S* ^2^ = *I*, *S* ^−1^ = *S*, *S* ^2*j*^ = *I*, and *S* ^2*j*+1^ = *S*, *j* ≥ 1. Using the replacement *B*_*k*_ = 9*X*_*k*_ + 11*X*_*k*_^3^, we have

(22) Xk+1−S=2I+15Xk2+3Xk4Bk−1−S=2I+15Xk2+3Xk4−SBkBk−1=2I+15Xk2+3Xk4−9SXk−11SXk3Bk−1=−−2S−15SXk2−3SXk4+9Xk+11Xk3 ×S−1Bk−1=Xk−S32I−3SXkS−1Bk−1.

Now, using any matrix norm from both sides of (22), we attain

(23) $$\begin{array}{c} \left\|X_{k+1}-S\right\|\leq \left(\left\|B_{k}^{-1}\right\|\left\|S^{-1}\right\|\left\|2I-3SX_{k}\right\|\right){\left\|X_{k}-S\right\|}^{3}. \end{array}$$

This reveals the cubical rate of convergence for the new method (12). The proof is complete.

It should be remarked that the reciprocal iteration obtained from (12) is also convergent to the sign matrix (3) as follows:

(24) $$\begin{array}{c} X_{k+1}=\left(9X_{k}+11X_{k}^{3}\right){\left[2I+15X_{k}^{2}+3X_{k}^{4}\right]}^{-1}, \end{array}$$

where *X*_0_ = *A*. This is named PM2. Similar convergence results as the ones given in Theorems 2-3 hold for (24).

A scaling approach to accelerate the beginning phase of convergence is normally necessary since the convergence rate cannot be seen in the initial iterates. Such an idea was discussed fully in [7] for Newton's method. An effective way to enhance the initial speed of convergence is to scale the iterates prior to each iteration; that is, *X*_*k*_ is replaced by *μ*_*k*_ *X*_*k*_. Subsequently, we can present the accelerated forms of our proposed methods as follows:

(25) $$\begin{array}{c} X_{0}=A,\\ \mu_{k}=\text{is}\;\text{the}\;\text{scaling}\;\text{parameter}\;\text{computed}\;\text{by}\;\left(27\right),\\ X_{k+1}=\left(2I+15\mu_{k}^{2}X_{k}^{2}+3\mu_{k}^{4}X_{k}^{4}\right){\left[9\mu_{k}X_{k}+11\mu_{k}^{3}X_{k}^{3}\right]}^{-1}, \end{array}$$

or
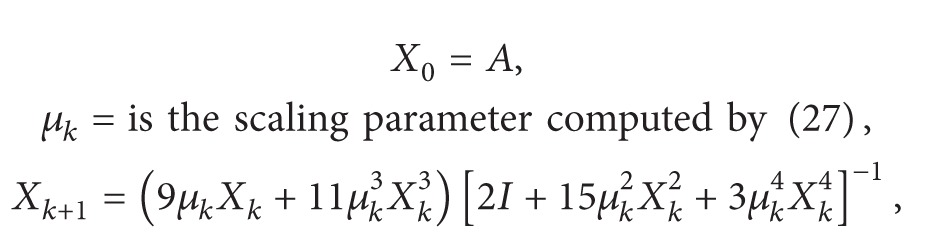
(26)

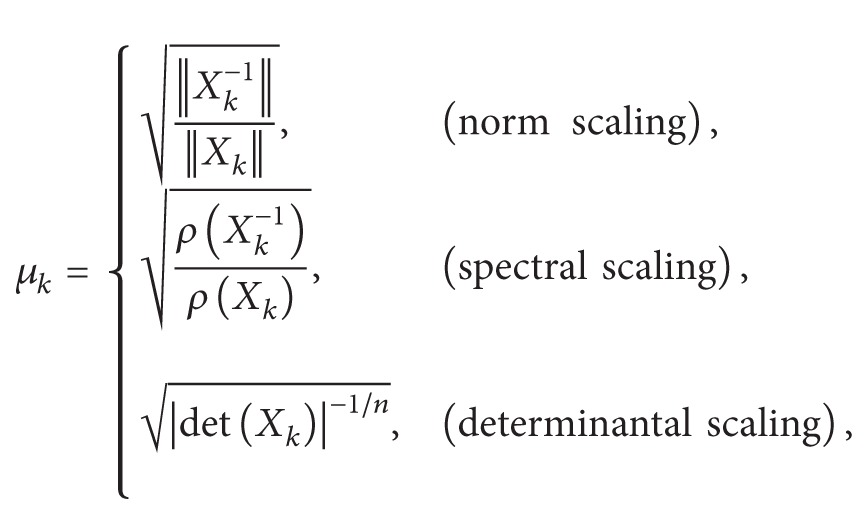
(27)where lim_*k*→*∞*_ *μ*_*k*_ = 1 and lim_*k*→*∞*_ *X*_*k*_ = *S*. The different scaling factors for *μ*_*k*_ in (27) are borrowed from Newton's method. For this reason it is important to show the behavior of the accelerator methods (25)-(26) and this will be done in the next section.

## 3. Numerical Examples

In this section, the results of comparisons in terms of number of iterations and the residual norms have been reported for various matrix iterations. We compare PM1 and PM2 with (6) denoted by NM and (8) denoted by HM. The programming package Mathematica [8] is used throughout this section. In Tables 1 and 2, IT stands for the number of iterates.

Note that the computational order of convergence for matrix iterations in finding *S* can be estimated by [9]

(28) $$\begin{array}{c} \rho =\frac{\log\left(\left\|X_{k+1}^{2}-I\right\|/\left\|X_{k}^{2}-I\right\|\right)}{\log\left(\left\|X_{k}^{2}-I\right\|/\left\|X_{k-1}^{2}-I\right\|\right)}, \end{array}$$

where *X*_*k*−1_, *X*_*k*_, and *X*_*k*+1_ are the last three approximations.

**Example 4 .**

**Figure 3:**
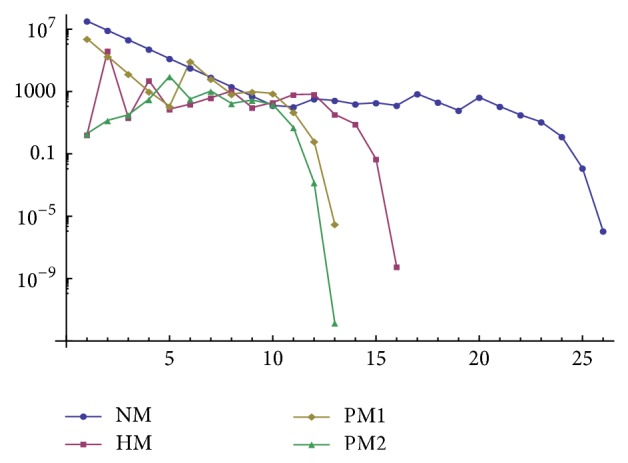
Convergence history versus number of iterations for different methods in Example 4.

In this example, we compare the methods for the following 500 × 500 complex matrix:

n = 500; SeedRandom[123];

A = RandomComplex[{-100 - I, 100 + I},{n,n}];

We apply here double precision arithmetic with the stop termination *R*_*k*+1_ = ‖*X*_*k*+1_^2^ − *I*‖_*∞*_ ≤ 10^−5^. Results are given in Figure 3.

**Example 5 (academic test).**

We compute the matrix sign for the following complex test problem:

(29) $$\begin{array}{c} A=\left(\begin{array}{cccc} 0 & 10 & i & 7+i\\ 7 & -5 & 6 & -5\\ 0 & 60 & -2 & 9\\ 0 & 5 & 9 & i \end{array}\right), \end{array}$$

where
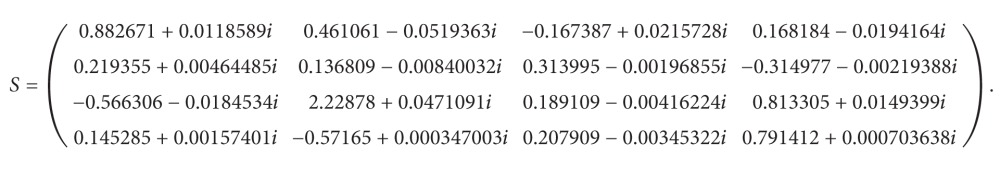
(30)
We apply here 600-digit fixed point arithmetic in our calculations with the stop termination *R*_*k*+1_ = ||*X*_*k*+1_^2^ − *I*||_*∞*_ ≤ 10^−150^. The results for this example are illustrated in Table 1. We report the COCs in *l*_*∞*_.

Iterative schemes PM1 and PM2 are evidently believed to be more favorable than the other compared methods due to their fewer number of iterations and acceptable accuracy. Hence, the proposed methods with properly chosen initial matrix *X*_0_ can be helpful in finding the sign of a nonsingular complex matrix.

**Example 6 .**

Here we rerun Example 5 using the scaling approaches (27) with the stop termination *R*_*k*+1_ = ||*X*_*k*+1_^2^ − *I*||_*∞*_ ≤ 10^−100^. The results for this example are illustrated in Table 2. We used the determinantal scaling for all compared methods. The numerical results uphold the theoretical discussions of Section 2.

A price paid for the high order convergence is the increased amount of matrix multiplications and inversions. This is a typical consequence. However the most important advantage of the presented methods in contrast to the methods of the same orders, such as (8), is their larger attraction basins. This superiority basically allows the new methods to converge to a required tolerance in one lower iteration than their same order methods. Hence, studying the thorough computational efficiency index of the proposed methods may not be an easy task and it must be pursued experimentally. In an experimental manner, if the costs of one matrix-matrix product and one matrix inversion are unity and 1.5 of unity, respectively, then we have the following efficiency indices for different methods: *E*_(6)_ = 2^1/(14(1)+14(1.5))^≃1.020, *E*_(8)_ = 3^1/(9(3)+9(1.5))^≃1.027, and *E*_(12)_ = 3^1/(8(4)+8(1.5))^≃1.025. Note that for Newton's method we have one matrix-matrix product per cycle due to the computation of stopping criterion. Other similar computations for efficiency indices for different examples show similar behaviors to the above mentioned one.

## 4. Summary

Matrix functions are used in many areas of linear algebra and arise in numerous applications in science and engineering. The function of a matrix can be defined in several ways, of which the following three are generally the most useful: Jordan canonical form, polynomial interpolation, and finally Cauchy integral.

In this paper, we have focus on iterative methods for this purpose. Hence, a third order nonlinear equation solver has been employed for constructing a new method for *S*. It was shown that the convergence is global via attraction basins in the complex plane and the rate of convergence is cubic. Furthermore, PM2 as the reciprocal of the method PM1 with the same convergence properties was proposed. The acceleration of PM1 and PM2 via scaling was also illustrated simply.

Finally some numerical examples in both double and multiple precisions were performed to show the efficiency of PM1 and PM2. Further researches must be forced to extend the obtained iterations for computing polar decompositions in future studies.

## Figures and Tables

**Table 1:** Results of comparisons for Example 5 using *X*_0_ = *A*.

Methods	NM	HM	PM1	PM2
IT	14	9	8	8
*R*_*k*+1_	1.41584 × 10^−249^	1.0266 × 10^−299^	2.5679 × 10^−298^	1.45091 × 10^−337^
ρ	1.99077	3	3	3

**Table 2:** Results of comparisons for Example 6 using *X*_0_ = *A*.

Methods	NM	HM	PM1	PM2
IT	10	7	6	6
*R*_*k*+1_	5.7266 × 10^−155^	5.80819 × 10^−203^	8.38265 × 10^−153^	1.55387 × 10^−143^
ρ	2.00228	3.00001	3.00015	3
